# Supplementary figures and images for: Opposing effects of acellular and whole cell pertussis vaccines on Bordetella pertussis biofilm formation, Siglec-F+ neutrophil recruitment and bacterial clearance in mouse nasal tissues
Source: bioRxiv. 2024 Jan 25:2024.01.23.576795. Preprint. [Version 1] doi: 10.1101/2024.01.23.576795 (PMC10849580; doi:10.1101/2024.01.23.576795)

**A**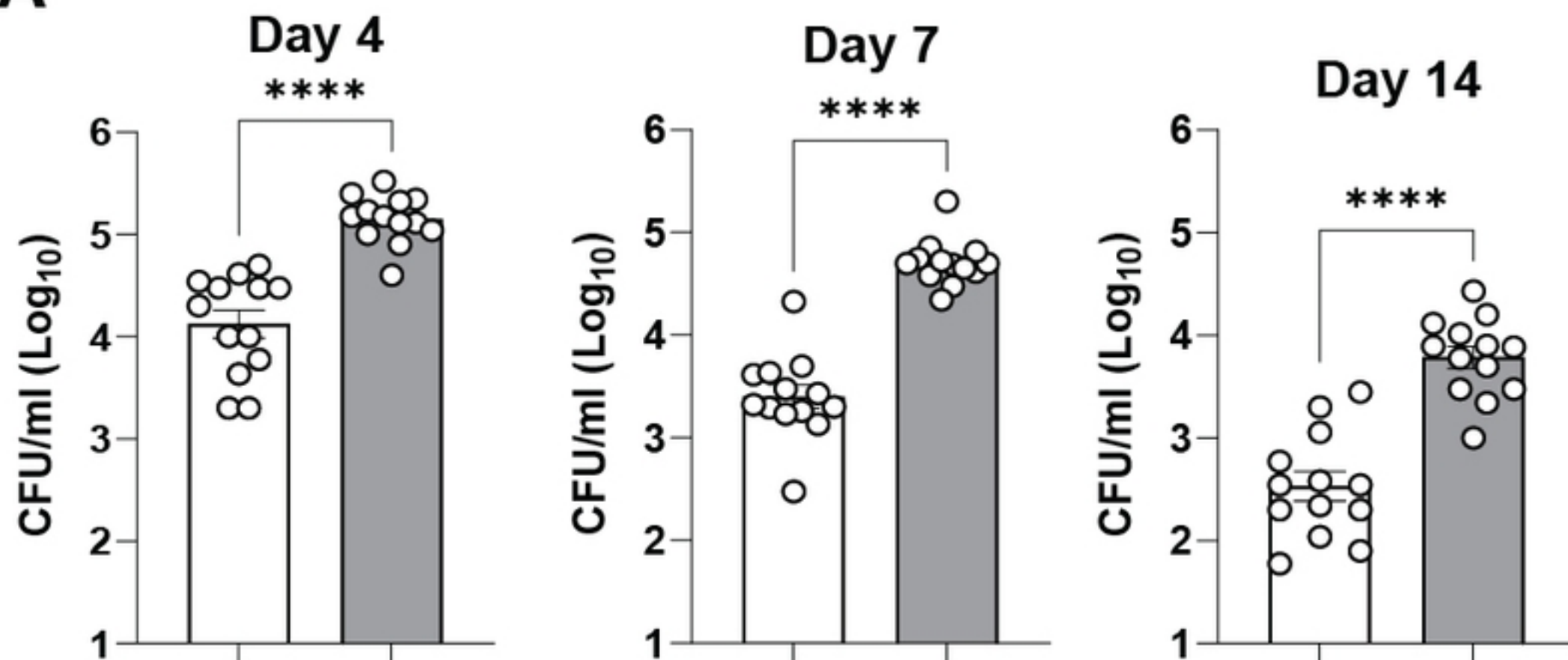**B**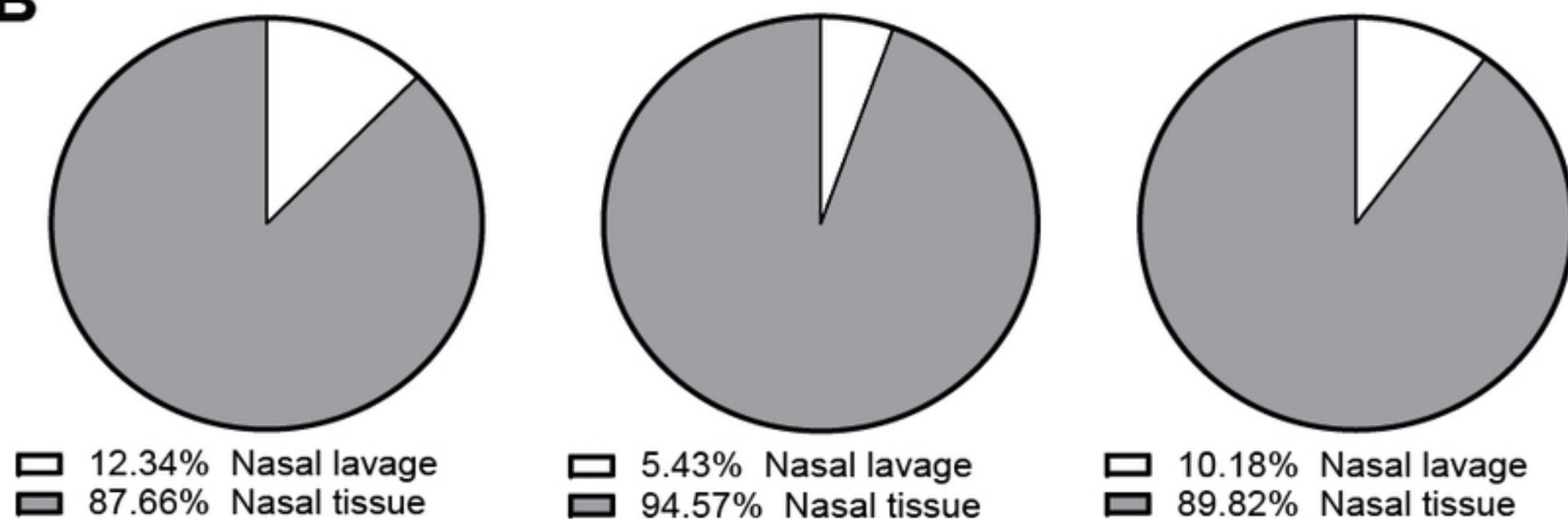

Figure S1

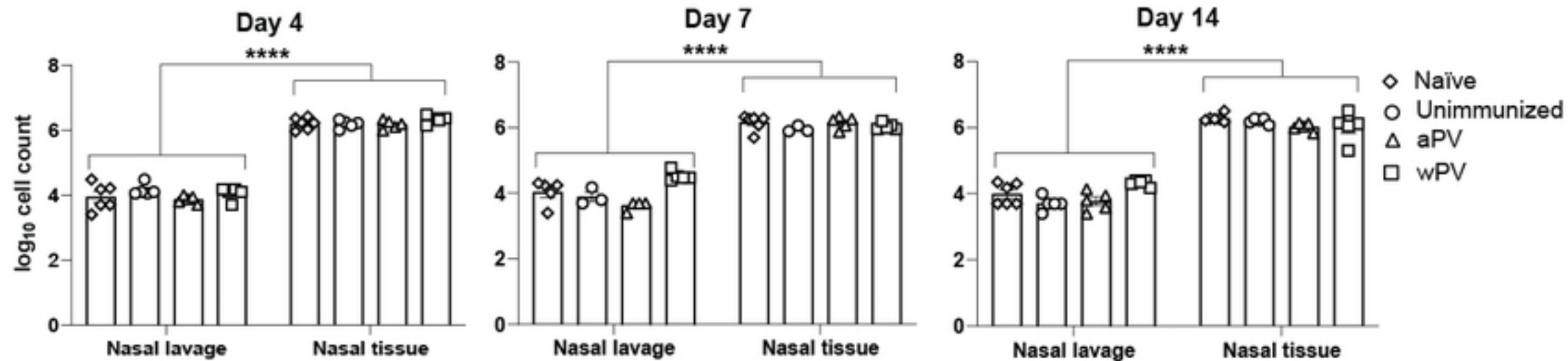

Figure S2

Supplement: Supplement 1 — Fig. S1. Nasal lavage alone underrepresents Bp burden in the nasal cavity. (A) Bp CFUs in the nasal lavage and nasal septum of unimmunized mice challenged with Bp and harvested at days 4, 7, and 14 post challenge. Data shown as mean ± SEM of 3 different experiments (N=13). CFUs in nasal lavage compared to the nasal associated tissue were determined by an unpaired t-test. ****P<0.0001. (B) Percentages of total bacteria that was collected in the NL compared to the bacterial burden in the NT. Fig. S2. Nasal lavage largely underrepresents the cellular responses in the nasal cavity. Following nasal lavage and nasal tissue collection and processing, total cell counts were determined for flow cytometry. Mean ± SEM (N= 5–6). ****P<0.0001. [file NIHPP2024.01.23.576795v1-supplement-1.pdf]
